# Association between quality of governance, antibiotic consumption, and antimicrobial resistance: an analysis of Italian regions

**DOI:** 10.1186/s13756-023-01337-6

**Published:** 2023-11-21

**Authors:** Andrea Maugeri, Martina Barchitta, Antonella Agodi

**Affiliations:** Department of Medical and Surgical Sciences and Advanced Technologies “GF Ingrassia”, University of Catania, Via S. Sofia 87, 95123 Catania, Italy

**Keywords:** Antimicrobial resistance, Antibiotic consumption, Antibiotic, Governance, Government, Italy, Regional differences

## Abstract

**Background:**

Emerging research has provided evidence suggesting the potential influence of governance on the development and spread of antimicrobial resistance (AMR), accounting for significant disparities observed both between and within countries. In our study, we conducted an ecological analysis to investigate the relationship between governance quality, antibiotic consumption, and AMR across Italian regions.

**Methods:**

By leveraging data from three distinct sources at the regional level, we compiled a comprehensive dataset comprising: AMR proportions for three specific pathogen-antibiotic combinations in the year 2021, antibiotic consumption data for systemic use in the year 2020, and the 2021 European Quality of Government Index (EQI) and its corresponding pillars. Employing mediation analysis, we investigated the potential mediating role of antibiotic consumption in the association between the EQI and an average measure of AMR.

**Results:**

Our analysis revealed substantial variation in the percentages of AMR across different regions in Italy, demonstrating a discernible North-to-South gradient concerning both antibiotic usage and governance quality. The EQI exhibited a statistically significant negative correlation with both antibiotic consumption and AMR percentages, encompassing both specific combinations and their average value. Regions characterized by higher levels of governance quality consistently displayed lower values of antibiotic consumption and AMR, while regions with lower governance quality tended to exhibit higher levels of antibiotic use and AMR. Furthermore, we observed a significant total effect of the EQI on average AMR (β = − 0.97; CI − 1.51; − 0.43). Notably, this effect was found to be mediated by antibiotic consumption, as evidenced by a significant indirect effect (β = − 0.89; CI − 1.45; − 0.32).

**Conclusions:**

These findings draw attention to the regional disparities observed in AMR levels, antibiotic consumption patterns, and governance quality in Italy. Our study also highlights the mediating role of antibiotic consumption in the relationship between governance quality and AMR. This underscores the significance of implementing focused interventions and policies aimed at improving governance quality and promoting responsible antibiotic use.

**Supplementary Information:**

The online version contains supplementary material available at 10.1186/s13756-023-01337-6.

## Background

Antimicrobial resistance (AMR) has emerged as a pressing global health threat, undermining the effectiveness of antibiotics and posing significant challenges to healthcare systems worldwide [1–4]. The overuse and misuse of antibiotics have been identified as key drivers of AMR, leading to reduced treatment options and increased morbidity and mortality from resistant infections [5–7]. To combat this growing crisis, it is crucial to understand the factors influencing antibiotic consumption patterns and the subsequent development of AMR [8–13].

While numerous studies have focused on individual-level determinants of antibiotic use [14–16], there is a growing recognition of the role played by broader contextual factors, including governance, in shaping antibiotic consumption practices and AMR [6, 7, 11–13, 17]. Governance refers to the processes, mechanisms, and institutions through which decisions are made, implemented, and monitored [18]. Effective governance in the healthcare sector can have a profound impact on healthcare policies, guidelines, and practices, including the appropriate use of antibiotics [19–22]. Quality of governance, characterized by transparency, accountability, regulatory frameworks, and stakeholder engagement, is increasingly recognized as a critical determinant of health system performance and outcomes [19–22]. Recent research has presented evidence highlighting the critical role of poor governance in driving AMR and accounting for the substantial variation observed among countries worldwide [6, 11–13, 17]. The impact of governance encompasses a range of multifaceted aspects, including regulatory frameworks, healthcare policies, surveillance systems, and stakeholder engagement [19–22]. Collectively, these factors shape the overall effectiveness of strategies aimed at mitigating AMR and exert influence over the trajectory of AMR development and dissemination. Therefore, gaining a deep understanding of the complex interplay among governance, antibiotic consumption, and AMR is crucial for formulating comprehensive interventions and policies that address the underlying systemic drivers of AMR while promoting responsible antibiotic use.

Despite this imperative, the association between governance and both antibiotic consumption and AMR has received limited attention, particularly at the local and national levels. Our study focused on Italy, a country that faces significant challenges related to the overuse of antibiotics and high AMR rates. The healthcare landscape in Italy is characterized by variations in healthcare practices, governance structures, and patient populations [23–26], presenting a unique opportunity to investigate the interplay between the quality of governance, antibiotic consumption, and AMR. The National Report on Antibiotic Use in Italy, as released by the Italian Medicines Agency, reveals an increasing north-to-south gradient among Italian regions. This gradient hints at possible discrepancies in attitudes, behaviours, and practices related to antibiotic consumption [27]. Regional differences in antibiotic consumption patterns reflect the complex dynamics at play and underscore the need to examine the role of governance in shaping prescribing behaviours and AMR outcomes. A comparable north-to-south gradient becomes apparent when analysing rates of AMR based on surveillance data [28]. These variations in AMR highlight the importance of considering regional factors in understanding the spread and emergence of resistant pathogens. Exploring the link between governance quality, antibiotic consumption, and AMR at the regional level provides valuable insights into the contextual factors influencing AMR outcomes and can inform targeted interventions to mitigate the rise of resistance. By delving into these relationships, our study could contribute to filling crucial knowledge gaps in the field, offering a foundation for evidence-based policy-making and targeted interventions against AMR.

By conducting an analysis using datasets that include antibiotic consumption data, AMR surveillance data, and indicators of governance quality, our aim is to study potential connections between governance quality, variations in antibiotic use and the prevalence of AMR in Italy.

## Methods

### Study design

A cross-sectional analysis was conducted on ecological data obtained from 19 Italian regions and the two autonomous provinces of Bolzano and Trento. The dataset was constructed by integrating variables related to AMR percentages, antibiotic consumption, and governance indicators, which were sourced from three different data sources.

### Antimicrobial resistance data

We utilized regional-level data on AMR proportions in Italy for the year 2021. These data were provided by the Italian AMR surveillance project of the Istituto Superiore di Sanità (AR-ISS), which focuses on selected isolates and specific periods across the country [29]. For the year 2021, the region of Campania did not take part in the surveillance activities [28]. The AR-ISS operates through a well-connected network of local clinical laboratories, dedicated to testing the antimicrobial susceptibility of isolates from invasive infections (bacteraemia and meningitis) that represent both community-acquired infections and healthcare-associated infections. Significantly noteworthy is the remarkable increase in the number of sentinel laboratories in Italy, which surged to 138 by 2021. This provides extensive nationwide coverage, encompassing approximately 55.3%, with notable variations observed among different Italian regions [28] (Additional file 1). The resulting AMR proportions are also reported to the European Antimicrobial Resistance Surveillance Network (EARS-Net) of the European Centre for Disease Prevention and Control (ECDC) [30]. The AMR data for the year 2021 have been included in the AR-ISS 2022 report [28] and have been made accessible to the public via a dedicated platform [29]. In our analysis, we incorporated pathogen-antibiotic combinations that exhibit the greatest global burden [1] and for which regional-level data are accessible. Specifically, we used data on methicillin-resistant *Staphylococcus aureus* (MRSA), carbapenem-resistant *Klebsiella pneumoniae* (CRKP), and third-generation cephalosporin-resistant *Escherichia coli* (CREC). The percentage of methicillin resistance in *S. aureus* refers to the resistance to at least one antibiotic among oxacillin and cefoxitin. The percentage of carbapenem resistance in *K. pneumoniae* refers to the resistance to at least one antibiotic among imipenem and meropenem. The percentage of third-generation cephalosporin resistance in *E. coli* refers to the resistance to at least one antibiotic among cefotaxime, ceftazidime, and ceftriaxone [28]. In addition, an average measure of AMR was constructed. Specifically, we initially standardized the percentages for each pathogen-antibiotic combination using the z-score method. This process involves transforming the data to have a mean of 0 and a standard deviation of 1. Subsequently, we calculated the average AMR measure by taking the mean of the individual standardized values. By standardizing the individual AMR proportions, the summary measure accounted for variations in resistance levels specific to each pathogen-antibiotic combination.

### Antibiotic consumption

We collected data on the consumption of antibacterials for systemic use (Anatomical Therapeutic Chemical [ATC] group J01) at the regional level for the year 2020, as well as the ratio between broad- and narrow-spectrum antibiotics. The inclusion of data from the year prior to the collection of AMR data is driven by the recognition of the potential influence of antibiotic consumption on AMR proportions. By considering the antibiotic consumption data from the previous year, we can evaluate the potential impact of this factor on the observed AMR proportions. To obtain information on antibiotic consumption, we referred to the 2020 National Report on Antibiotic use in Italy, which was published by the Italian Medicines Agency and made accessible through the Medicines Utilisation Monitoring Centre in 2022 [31]. Antibiotic consumption, in this context, refers to both reimbursed medications and purchases made by public healthcare facilities. It was measured in terms of standard doses, specifically daily defined doses (DDD), per 1,000 individuals per day. It is important to note that DDD represents the number of doses required for one day of adult treatment for each specific medication [31].

### Governance indicators

We used data on the European Quality of Government Index (EQI) and its pillars, which comprehensively assess citizens' perceptions and experiences regarding corruption, quality, and impartiality in essential public services [32]. The latest edition of the EQI, published in 2021, involved an extensive survey conducted across all 27 European Union member states, encompassing 208 regions at the Nomenclature of territorial units for statistics (NUTS) 1 or NUTS2 level. With a remarkable participation of over 129,000 respondents, this survey stands as the largest endeavour to date in measuring government quality perceptions in the EU [33]. The primary focus of the survey was to capture citizens' perspectives on public healthcare, education, and law enforcement, aiming to encapsulate the multidimensional nature of quality of government. This multidimensional concept comprises impartiality, quality in public service delivery, and the absence of corruption [33].

The EQI serves as a composite indicator, amalgamating data from seventeen survey items to estimate the level of quality of government at the regional level. The aggregation process involved standardizing and centering the data using World Governance Indicators measures pertaining to corruption, government effectiveness, rule of law, and voice and accountability. To ensure representativeness, the study incorporated weighting and post-stratification techniques. The regional data were combined, resulting in a singular EQI score for each region. This composite score, expressed as numerical value, serves as a comprehensive indicator of overall regional performance based on citizens' perceptions and experiences. Higher EQI scores indicate a better perceived quality of government, including lower corruption levels, heightened impartiality, and superior quality in public service delivery. Conversely, lower EQI scores indicate areas where improvements in these dimensions are necessary [33].

### Statistical analysis

Descriptive analysis was performed on the individual variables to summarize their characteristics. The data concerning antibiotic consumption, the EQI and its pillars, and the percentages of AMR for specific pathogen-antibiotic combinations were processed in their original form, as provided by their respective sources [28, 29, 31, 33]. Geospatial maps were created to visually depict the geographical distribution of the average AMR measure, antibiotic consumption, and the EQI.

We examined the relationships between the variables using bivariate analysis, employing Spearman's correlation coefficient. Subsequently, after verifying the statistical assumptions, we utilized simple linear regression models to evaluate the association between the EQI (as the independent variable) and both antibiotic consumption and the average AMR measure (as the dependent variables). In each model, the variables were included as continuous numeric variables.

Lastly, mediation analysis was performed to explore the mediating role of antibiotic consumption between the EQI and AMR. This approach provides a robust method to assess both the direct and indirect effects of the independent variable on the dependent variable, mediated through the proposed mediator. In general, based on the framework proposed by Baron and Kenny [34], there are two paths to the dependent variable. In our analysis, the independent variable (EQI) must predict the dependent variable (the average AMR measure), and the independent variable must predict the mediator (antibiotic consumption). Mediation was tested through three linear regressions: (i) independent variable predicting the dependent variable; (ii) independent variable predicting the mediator; (iii) independent variable and mediator predicting the dependent variable. Accordingly, the following conditions must be met to support mediation: (i) the independent variable significantly predicts the dependent variable in the first regression, (ii) the independent variable significantly predicts the mediator in the second regression; (iii) the mediator significantly predicts the dependent variable while controlling for the independent variable. In our study, we accounted for these assumptions by following the methodology outlined by Preacher and Hayes [35]. Firstly, the analysis examined the relationship between the EQI and AMR, establishing the total effect. Secondly, the association between the EQI and antibiotic consumption was assessed, establishing the predictor-mediator relationship. Thirdly, the relationship between the mediator (antibiotic consumption) and the dependent variable (average AMR measure) was evaluated, controlling for the EQI. This step determined the mediator-outcome relationship. Finally, the indirect effect of the EQI on AMR through antibiotic consumption was assessed. To obtain reliable estimates, bias-corrected and accelerated bootstrap confidence intervals (CIs) were utilized. All statistical analyses were performed using the SPSS software (version 29.0, SPSS, Chicago, IL), with a significance level α of 0.05.

## Results

**Fig. 1:**
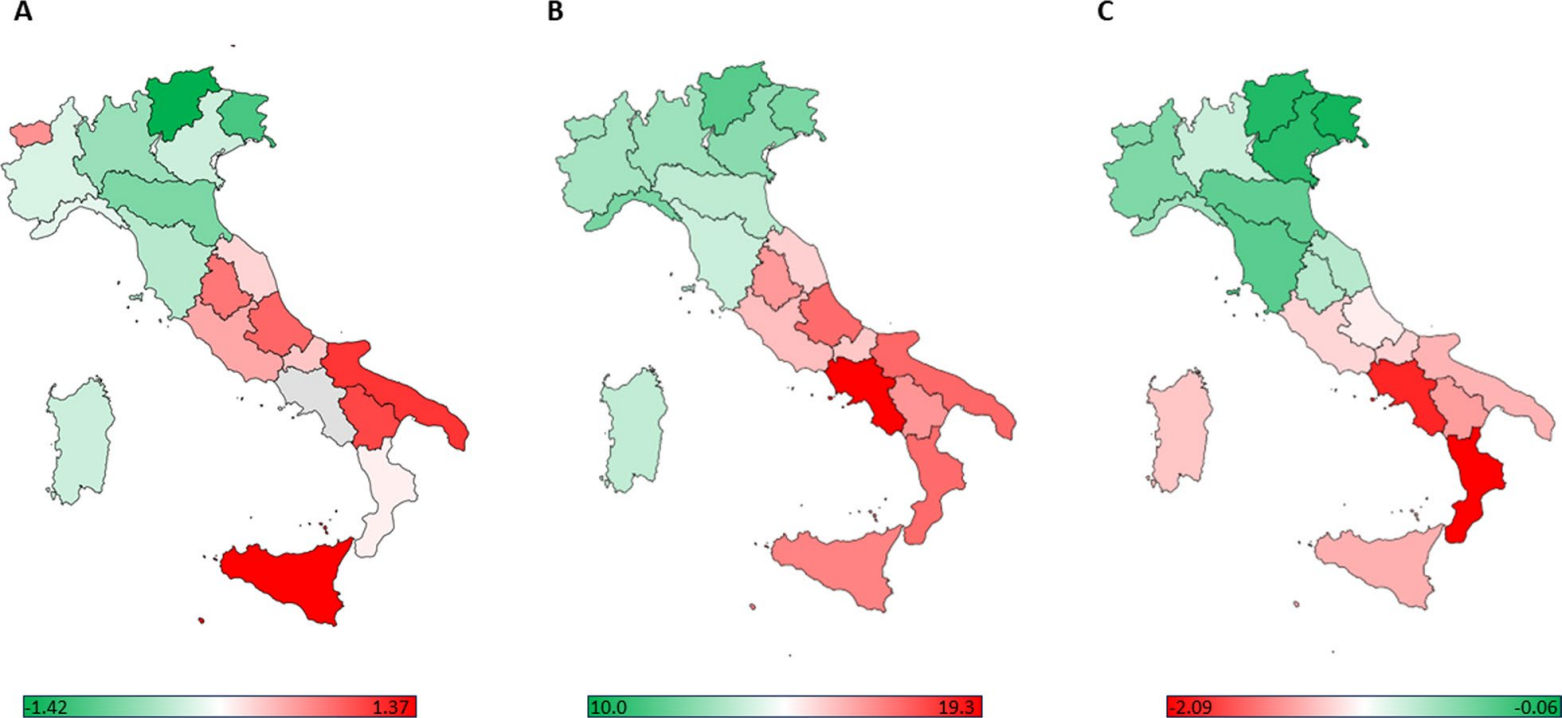
Geographical distribution of antimicrobial resistance (AMR), antibiotic consumption, and the European Quality of Government Index (EQI) across Italian regions in 2021. The maps depict the following: **A** Average AMR measure, calculated as the mean of AMR proportions standardized by pathogen and antibiotic class; **B** Antibiotic consumption reported as DDD per 1,000 individuals per day; **C** The EQI, a comprehensive indicator that reflects the overall regional performance based on citizens' perceptions and experiences. Higher EQI scores indicate a higher perceived quality of government, characterized by lower levels of corruption, increased impartiality, and superior quality in public service delivery

In the year 2021, AMR data at the regional level are accessible for eighteen regions and two autonomous provinces (Bolzano and Trento). Figure 1A illustrates the average measure of AMR, obtained by averaging the standardized resistance proportions for each pathogen-antibiotic combination. Regional variations in the resistance percentages for the three examined pathogen-antibiotic combinations are reported in the Additional file 2. A degree of variation in AMR percentages across the Italian regions is widely observed. Specifically, for MRSA, the percentages range from 7.4% in the autonomous province of Bolzano to 80.0% in Abruzzo. Regarding CRKP, the percentages vary from 5.1% in the autonomous province of Bolzano to 66.1% in Basilicata. As for CREC, the percentages range from 11.9% in the autonomous province of Bolzano to 38.2% in Apulia. These findings show disparities in AMR levels across various regions in Italy. Specifically, Sicily, Apulia, and Basilicata exhibited the highest average AMR values, indicating elevated levels of AMR. In contrast, the autonomous provinces of Bolzano and Trento, and Friuli-Venezia Giulia demonstrated the lowest average AMR values, indicating comparatively lower AMR levels in these regions.

Significant variability among the regions was also observed regarding antibiotic consumption, exhibiting a clear increasing gradient from northern to southern Italy (Fig. 1B). Apart from Campania (which was not included in the following analysis but reported a value of 19.3 DDD per 1,000 individuals per day), the highest values were observed in Apulia (17.0), Abruzzo (16.9), and Calabria (16.9). In contrast, the lowest values were reported in the autonomous province of Bolzano (8.0), Liguria (10.7), and Friuli Venezia Giulia (10.7). When considering the ratio between broad- and narrow-spectrum antibiotic use, regional variation has been evident: Liguria, Sardinia, and Lazio have exhibited the highest values in this regard, indicating a higher proportion of broad-spectrum antibiotic utilization. Conversely, Emilia Romagna, Friuli Venezia Giulia, and Tuscany have shown the lowest values. However, these regional disparities did not follow a distinct or consistent geographical gradient across the regions.

A pronounced North-to-South gradient was instead apparent in terms of governance quality, evaluated through the EQI. In general, the regions in Northern Italy reported higher levels of governance quality, as reflected by higher EQI scores, while the scores were comparatively lower for the Central and Southern regions of Italy. Specifically, the highest EQI scores were observed in the autonomous provinces of Trento, Friuli Venezia Giulia, and Veneto, while the lowest scores were reported in Calabria, Campania, Basilicata, and Sicily (Fig. 1C). Comparable patterns were observed for the pillars and sub-pillars of the EQI, indicating similar gradients (Additional file 3).

Expanding on this scenario, we conducted an analysis to examine the correlation between the EQI and its respective pillars and sub-pillars, along with antibiotic consumption and AMR data. The EQI displayed a statistically significant negative correlation with both antibiotic consumption and AMR, for both the specific combinations and their average measure. However, there was no significant correlation observed between the EQI and the ratio of broad-spectrum to narrow-spectrum antibiotics. Comparable results were obtained when considering the pillars and sub-pillars of the EQI, except for the experienced corruption, which did not show a significant correlation (Table 1).

**Table 1:** The relationships between governance indicators, antibiotic use, and antimicrobial resistance (AMR)

Governance indicators	Antibiotic consumption	Broad vs Narrow Spectrum Antibiotics	MRSA	CRKP	CREC	Aggregate AMR measure
EQI	− 0.791***	− 0.313	− 0.481*	− 0.701**	− 0.660**	− 0.720***
Quality	− 0.786***	− 0.386	− 0.472*	− 0.687**	− 0.657**	− 0.713***
Impartiality	− 0.755***	− 0.310	− 0.432	− 0.642**	− 0.556*	− 0.646**
Corruption	− 0.830***	− 0.266	− 0.471*	− 0.665**	− 0.677**	− 0.734***
Experienced Corruption	− 0.487*	− 0.271	− 0.126	− 0.247	− 0.280	− 0.322
Perceived Corruption	− 0.826***	− 0.249	− 0.517*	− 0.707**	− 0.734***	− 0.765***

Results are reported as Spearman's correlation coefficients of the bivariate relationships *p < 0.05; **p < 0.01; ***p < 0.001

*MRSA* methicillin-resistant *Staphylococcus aureus*, *CRKP* carbapenem-resistant *Klebsiella pneumoniae*, *CREC* third-generation cephalosporin-resistant *Escherichia coli*, *AMR* antimicrobial resistance; *EQI* European Quality of Government Index

**Fig. 2:**
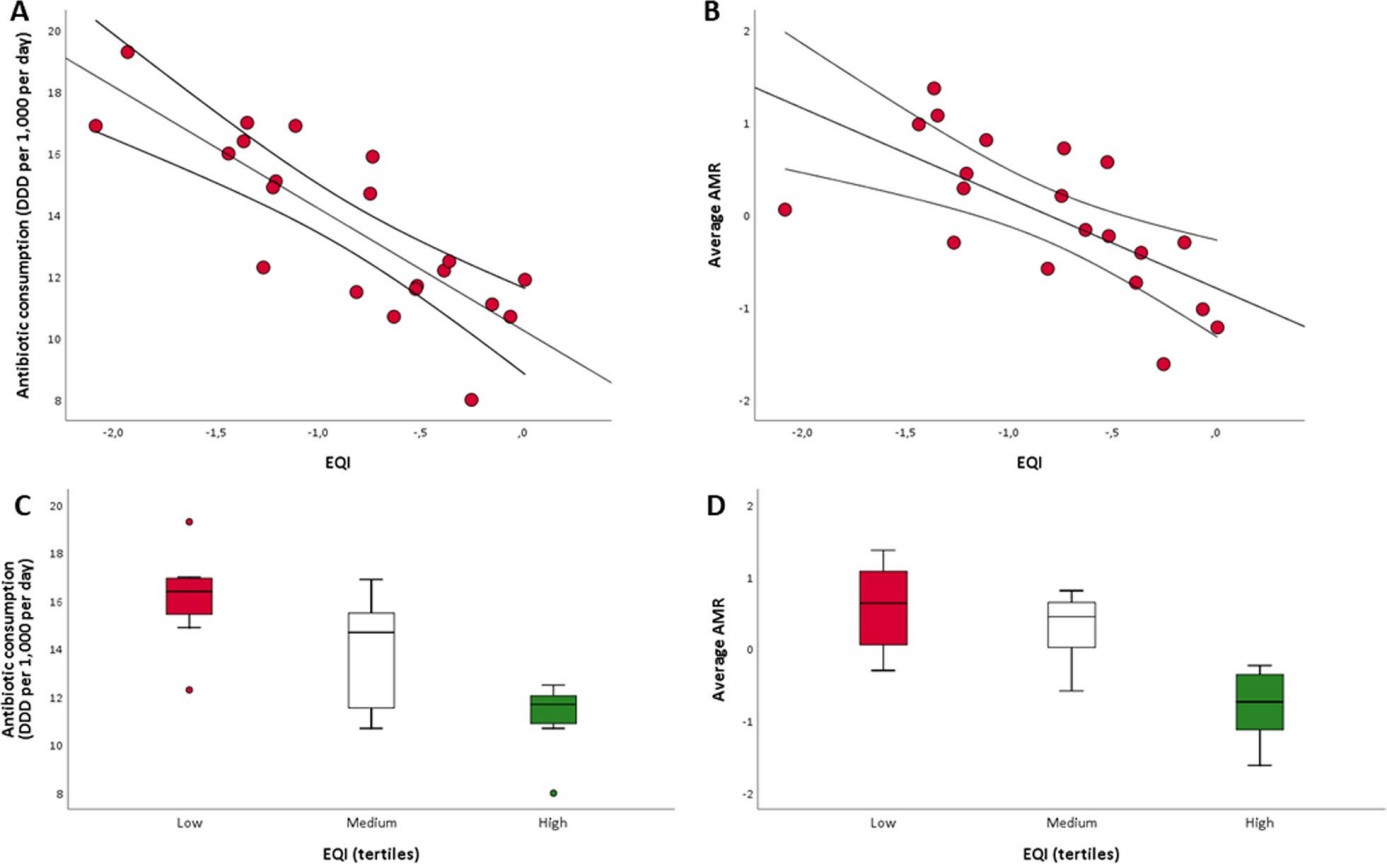
The relationships between the European Quality of Government Index (EQI), antibiotic consumption, and the average antimicrobial resistance (AMR). The scatter plots in (**A**) and (**B**) visually represent the linear relationships (with 95% confidence intervals) of the EQI with antibiotic consumption and average AMR, respectively. The box plots in (**C**) and (**D**) illustrate the differences in antibiotic consumption and average AMR across tertiles of the EQI

Hence, our attention was directed towards exploring the association of the EQI with antibiotic consumption and the average AMR measure (Fig. 2A, B). Through the application of simple linear regression models, a distinct inverse relationship between the EQI and both antibiotic consumption and average AMR became evident (β = − 3.96; SE = 0.64; *p* < 0.001 and β = − 0.97; SE = 0.25; *p* = 0.001, respectively). Furthermore, by stratifying the regions into tertiles based on the EQI distribution, we categorized them into those with low governance quality (1st tertile), moderate quality (2nd tertile), and high quality (3rd tertile). Upon comparing these groups (Fig. 2C, D), the differences in antibiotic consumption and AMR become even more pronounced, aligning with the varying levels of governance quality.

**Fig. 3:**
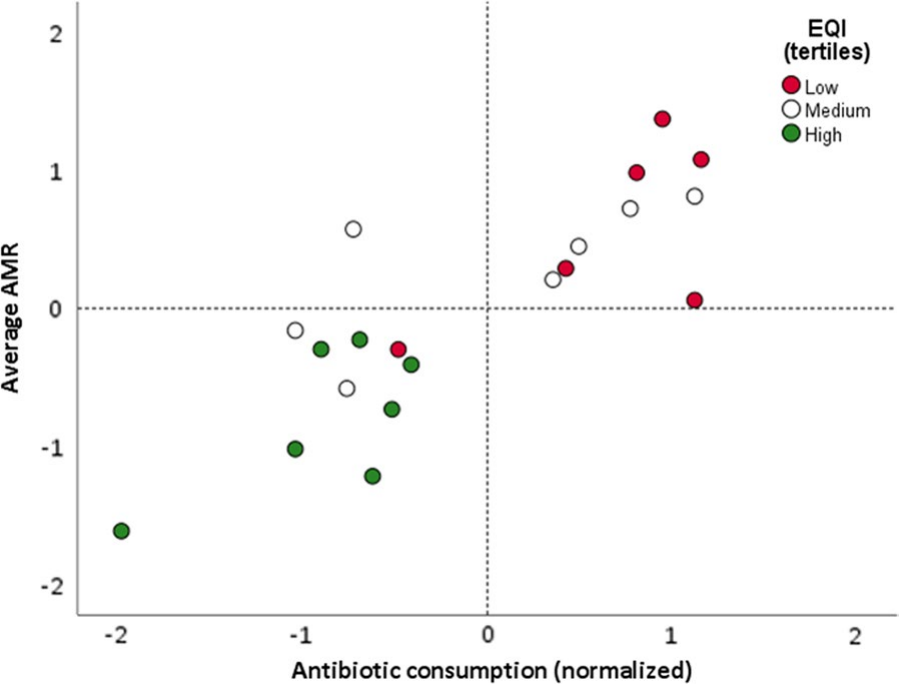
Quadrant analysis chart of the relationship between the European Quality of Government Index (EQI), antibiotic consumption, and the average antimicrobial resistance (AMR). The quadrant analysis chart visualizes the relationship between antibiotic consumption and average AMR in a two-dimensional space divided into four quadrants. The regions are color-coded according to their tertiles of the EQI. In this chart, the bottom left quadrant represents the most favourable scenario, characterized by regions with low antibiotic consumption and low average AMR. Conversely, the top right quadrant corresponds to the least desirable scenario, indicating regions with high antibiotic consumption and high average AMR

**Fig. 4:**
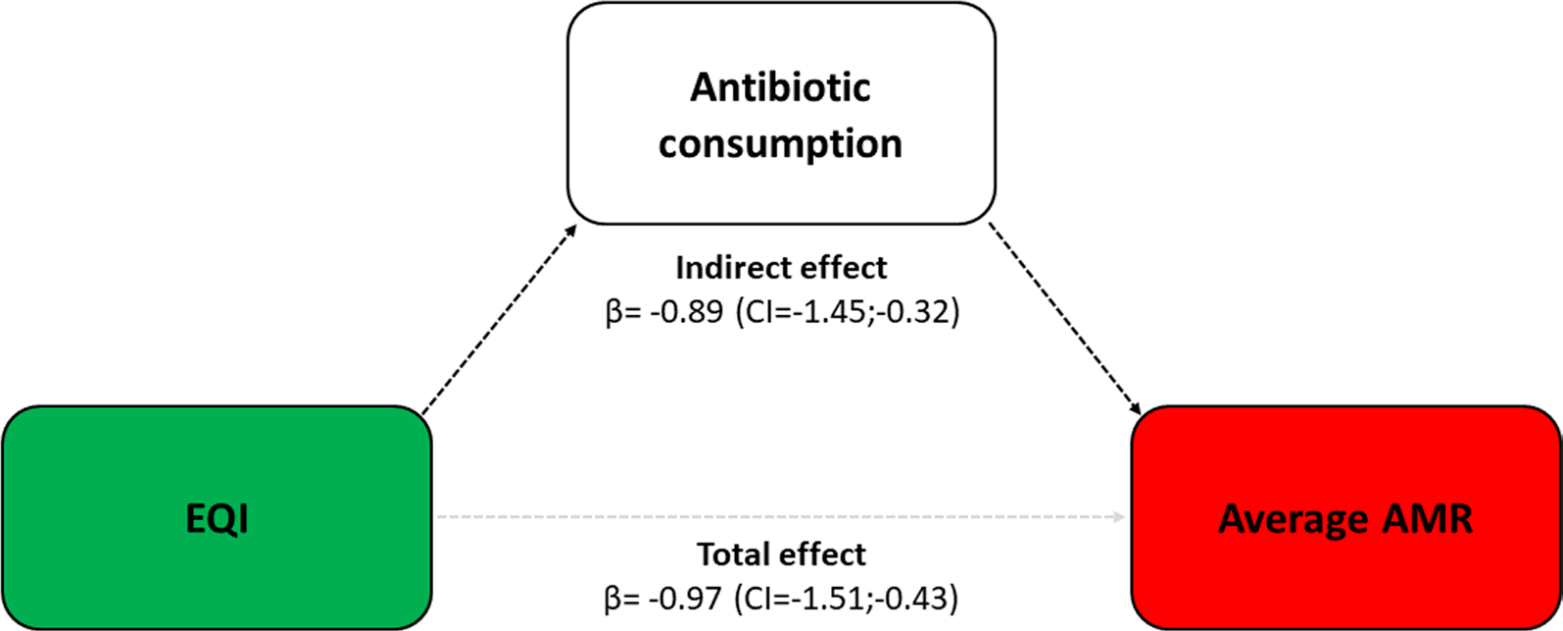
Mediation analysis of the relationship between the European Quality of Government Index (EQI), antibiotic consumption, and the average antimicrobial resistance (AMR). The diagram highlights the total effect of the EQI on average AMR and the indirect effect mediated through antibiotic consumption

By visually representing antibiotic consumption and average AMR on a graph (Fig. 3), a quadrant analysis was conducted. The bottom left quadrant was identified as the most favourable scenario, characterized by low antibiotic consumption and low value of average AMR. On the other hand, the top right quadrant represented the least desirable scenario, indicating high antibiotic consumption and high average AMR. Remarkably, all regions characterized by high governance quality were situated in the bottom left quadrant. Conversely, regions with low governance (except for one) predominantly occupied the top right quadrant. Thus, the EQI exhibited a dual association with both antibiotic consumption and the average AMR measure, prompting us to conduct a mediation analysis with antibiotic use serving as the mediator (Fig. 4). Importantly, a significant total effect of the EQI on average AMR was observed (β = − 0.97; CI = − 1.51; − 0.43). However, this effect was mediated by antibiotic consumption, as indicated by a significant indirect effect (β = − 0.89; CI = − 1.45; − 0.32). More precisely, the absence of a significant relationship between the EQI and the average AMR measure once the mediator is taken into account (β = − 0.08; CI = − 0.74; 0.58) suggests a complete mediation.

## Discussion

To the best of our knowledge, this study is the first examining the relationships between governance quality and regional variations in antibiotic consumption and AMR across Italian regions. By adopting a regional perspective, we enhance our understanding of the factors that contribute to antibiotic use and AMR, specifically focusing on the level of quality, impartiality, and corruption in essential public services.

Through the analysis of surveillance data pertaining to specific pathogen-antibiotic combinations [28, 29], our initial findings revealed regional disparities in a comprehensive measure of AMR among Italian regions. This, therefore, constitutes a significant concern warranting investigation [36–38]. The high AMR level observed in several regions of Southern Italy underscores the substantial challenges in managing infections caused by resistant pathogens in these areas [39–50]. These findings suggest the need for targeted interventions and strategies to strengthen antimicrobial stewardship programs, enhance infection prevention and control measures, and optimize antibiotic prescribing practices in the affected regions [51–58]. Conversely, our study also reports the presence of regions, primarily concentrated in Northern Italy, which exhibit lower levels of AMR. These regions may serve as exemplars for successful strategies in containing AMR and should be regarded as models for best practices [59].

The observed regional disparities in AMR levels across Italian regions may be associated with variations in antibiotic consumption, which is a relationship that has been extensively reported and well-established [27, 31, 60–62]. Specifically, we reported data from the 2020 National Report on Antibiotic use in Italy [31], showing a North-to-South gradient in antibiotic consumption, with higher rates observed in the southern regions compared to the northern regions. The higher rates of antibiotic consumption in regions with elevated AMR levels suggest that excessive antibiotic use may contribute to the selection and spread of resistant bacteria [63–65]. In regions with higher antibiotic consumption, the likelihood of encountering resistant bacteria may be increased. There is in fact a consensus that the excessive use of antibiotics not only lacks clinical benefits but also presents a substantial public health risk by accelerating the development and dissemination of AMR [64, 66]. Moreover, the overuse of antibiotics may be often accompanied by inadequate prescribing practices, such as overprescribing, incorrect dosages, and prolonged treatment durations, which create favourable conditions for the emergence and proliferation of resistant strains [67–70].

On the other hand, our study provides further evidence that regions with lower AMR levels tend to exhibit lower rates of antibiotic consumption. It would be plausible to hypothesize that these regions exhibit lower levels of AMR also due to reduced antibiotic consumption [5–7].

In addition to the observed regional disparities in AMR and antibiotic consumption, our findings also report a North-to-South gradient in governance quality. The association between governance quality, as measured by the EQI, and both antibiotic consumption and AMR suggests the potential role of effective governance in addressing AMR. Regions characterized by lower governance quality exhibited elevated levels of both antibiotic consumption and AMR. This hypothesis was substantiated by the mediation analysis conducted in our study, which offered additional insights into the interplay between governance, antibiotic consumption, and AMR. The significant indirect effect of governance on AMR, mediated through antibiotic consumption, hints the influence of governance on antibiotic utilization, which subsequently affects AMR outcomes. These findings highlight the importance of comprehensive strategies that target governance issues to effectively address AMR [71–73]. Interventions aimed at improving governance structures, promoting evidence-based prescribing practices, enhancing surveillance systems, and implementing antimicrobial stewardship programs can significantly contribute to mitigating the impact of AMR [71–78]. By addressing governance-related factors, policymakers and healthcare stakeholders can create an environment that fosters responsible antibiotic use and strengthens the overall management of AMR.

Our findings seem in line with previous evidence from ecological analyses conducted at the global and European levels. Studies examining various indicators of governance, including voice and accountability, government effectiveness, regulatory quality, rule of law, and control of corruption, consistently demonstrate an inverse relationship with both antibiotic consumption and AMR [11–13, 17]. Effective governance supports the development and implementation of policies and programs aimed at promoting rational antibiotic use, improving infection prevention and control measures, and enhancing surveillance and monitoring of AMR [71–78]. Robust regulatory systems, strong enforcement mechanisms, and transparent accountability structures are vital components of good governance that facilitate coordinated and impactful actions against AMR [73, 74, 76, 77]. Conversely, poor governance characterized by weak regulatory systems, inadequate enforcement, and limited accountability can hinder progress in combating AMR. Insufficient or ineffective actions against AMR may stem from a lack of resources, limited coordination among stakeholders, and inadequate policies [73, 74, 76, 77]. Weak governance can also contribute to improper antibiotic use, such as overprescribing, inappropriate prescribing, and inadequate adherence to treatment guidelines [6, 17, 79]. These practices exacerbate the development and spread of AMR, posing a significant threat to public health. Additionally, weak governance can impede the allocation of resources necessary to address AMR effectively, including investment in research and development of new antibiotics and alternative treatment options [6, 17, 80]. Inadequate governance structures may fail to prioritize AMR as a critical public health issue, leading to limited funding and insufficient attention to implementing comprehensive strategies [6, 17].

While our study contributes to the growing body of evidence on the association between governance quality, antibiotic consumption, and AMR, it is important to acknowledge its limitations. Firstly, the study was based on ecological and cross-sectional data, which restricts our ability to establish causality. More specifically, the absence of a control group could potentially impact the reliability and validity of our findings. It makes it difficult to discern whether the observed outcomes are a direct result of the factors we are investigating or if they are influenced by unrelated variables. Secondly, our analysis centered around three pathogen-antibiotic combinations that pose significant public health challenges and for which regional-level data are accessible. In the context of our analysis, it would have provided valuable insights to have access to the ECDC composite index, which incorporates data on MRSA, vancomycin-resistant E. faecium and E. faecalis, third-generation cephalosporin-resistant Enterobacteriaceae, as well as carbapenem-resistant P. aeruginosa and A. baumannii.

When examining antibiotic consumption, we utilized data from the year 2020, which preceded the AMR proportion data for 2021. In contrast, for the EQI, we had access to 2021 data, as it was not available on an annual basis. The additional data on the EQI from the years 2010, 2013, and 2017 were not considered for the present analysis. Therefore, future longitudinal studies are warranted to explore the temporal relationship between governance, antibiotic use, and AMR. Furthermore, the limited dataset size constrained our ability to incorporate additional factors that may be involved in the observed relationships. Although antibiotic consumption plays a significant role as a mediator in the governance-AMR relationship, it is important to acknowledge the presence of other complex dynamics that may influence AMR outcomes. For instance, certain cultural dimensions, such as uncertainty avoidance and individualism, have been identified as social factors that contribute to AMR. Notably, these dimensions may exhibit diverse patterns among various regions within Italy. Addressing these factors in future studies will enable a more precise assessment of the intricate relationship between governance, antibiotic consumption, and AMR. Finally, the study focused on regional-level data and did not examine individual-level factors that may contribute to AMR. Future research should investigate the individual determinants of antibiotic consumption and AMR to gain a more comprehensive understanding of the underlying factors.

On the other hand, the study has some strengths. It utilized data from multiple sources, allowing for an analysis of the association between governance, antibiotic consumption, and AMR. By incorporating data from various dimensions, our study adopted a multidimensional approach that helps provide an understanding of the dynamics in play. Additionally, our study incorporated the EQI as a measure of governance quality, providing a multidimensional perspective on governance beyond the healthcare sector. This approach allows us to examine the broader context in which antibiotic consumption and AMR occur, considering factors such as corruption, public service quality, and impartiality.

## Conclusions

This ecological study contributes additional insights into the intricate interplay among governance, antibiotic consumption, and AMR. Italy, in particular, presents an authentic research environment owing to the significant variations in AMR prevalence, antibiotic consumption levels, and governance indices observed across its diverse regions. If regions characterized by high governance quality showed reduced antibiotic consumption and lower levels of AMR, contrastingly, regions marked by poorer governance exhibited elevated rates of both. Specifically, our findings suggest the mediating effect of antibiotic consumption in the relationship between governance quality and AMR. This aligns with earlier research findings indicating that governance can primarily impact antibiotic consumption, subsequently affecting AMR levels. However, the limitations of our study strongly advocate for additional longitudinal investigations that take into account individual-level factors and account for potential confounding variables. These efforts will provide a deeper understanding of the intricate mechanisms that underlie the connection between governance, antibiotic use, and AMR.

### Supplementary Information

**Additional file 1.** Regional coverage of surveillance data on antimicrobial resistance in 2021

**Additional file 2.** Percentages of AMR for specific pathogen-antibiotic combinations and the average AMR measure across Italian regions in 2021

**Additional file 3.** Pillars and sub-pillars of the European Quality of Government Index across Italian regions in 2021

## Data Availability

The datasets used and/or analysed during the current study are available from the corresponding author on reasonable request.

## Abbreviations

AMR: Antimicrobial resistance
AR-ISS: AMR surveillance project of the Istituto Superiore di Sanità
EARS-Net: European Antimicrobial Resistance Surveillance Network
ECDC: European Centre for Disease Prevention and Control
MRSA: Methicillin-resistant *Staphylococcus aureus*
CRKP: Carbapenem-resistant *Klebsiella pneumoniae*
CREC: Third-generation cephalosporin-resistant *Escherichia coli*
ATC: Anatomical Therapeutic Chemical
DDD: Defined Daily Dose
EQI: European Quality of Government Index
NUTS: Nomenclature of territorial units for statistics
CI: Confidence interval
