# Supplementary material for: Association between quality of governance, antibiotic consumption, and antimicrobial resistance: an analysis of Italian regions
Source: Antimicrob Resist Infect Control. 2023 Nov 21;12:130. doi: 10.1186/s13756-023-01337-6 (PMC10662482; doi:10.1186/s13756-023-01337-6)
Supplement: Supplementary file 1 — Additional file 1. Regional coverage of surveillance data on antimicrobial resistance in 2021 [file 13756_2023_1337_MOESM1_ESM.docx]

**ADDITIONAL FILES**

**Additional File 1. Regional coverage of surveillance data on antimicrobial resistance in 2021.**

| **Region** | **Regional coverage** |
| --- | --- |
| Piemonte | 36.9% |
| Valle d'Aosta | 97.1% |
| Liguria | 93.7% |
| Lombardia | 55.3% |
| Autonomous Province of Trento | 93.7% |
| Autonomous Province of Bolzano | 99.4% |
| Veneto | 78.1% |
| Friuli-Venezia Giulia | 68.2% |
| Emilia-Romagna | 95.1% |
| Toscana | 92.0% |
| Umbria | 92.9% |
| Marche | 36.3% |
| Lazio | 31.4% |
| Abruzzo | 23.0% |
| Molise | 48.0% |
| Campania | - |
| Puglia | 40.1% |
| Basilicata | 75.3% |
| Calabria | 20.7% |
| Sicilia | 64.4% |
| Sardegna | 18.2% |
