# Supplementary material for: Association between quality of governance, antibiotic consumption, and antimicrobial resistance: an analysis of Italian regions
Source: Antimicrob Resist Infect Control. 2023 Nov 21;12:130. doi: 10.1186/s13756-023-01337-6 (PMC10662482; doi:10.1186/s13756-023-01337-6)
Supplement: Supplementary file 2 — Additional file 2. Percentages of AMR for specific pathogen-antibiotic combinations and the average AMR measure across Italian regions in 2021 [file 13756_2023_1337_MOESM2_ESM.docx]

**ADDITIONAL FILES**

**Additional File 2. Percentages of AMR for specific pathogen-antibiotic combinations and the average AMR measure across Italian regions in 2021.**

| **Region** | **MRSA** | **CRKP** | **CREC** | **Average AMR measure** |
| --- | --- | --- | --- | --- |
| Piemonte | 32.9% | 31.7% | 19.3% | -0.23 |
| Valle d'Aosta | 27.1% | 54.2% | 31.3% | 0.57 |
| Liguria | 34.4% | 19.6% | 25.1% | -0.16 |
| Lombardia | 28.2% | 13.1% | 21.4% | -0.58 |
| Autonomous Province of Bolzano | 7.4% | 5.1% | 11.9% | -1.61 |
| Autonomous Province of Trento | 17.2% | 8.2% | 14.6% | -1.22 |
| Veneto | 30.2% | 22.4% | 23% | -0.30 |
| Friuli-Venezia Giulia | 24.7% | 12.8% | 13.3% | -1.02 |
| Emilia-Romagna | 23.1% | 8.3% | 22.6% | -0.73 |
| Toscana | 23.2% | 17.8% | 26.0% | -0.41 |
| Umbria | 42.5% | 45.8% | 30.2% | 0.72 |
| Marche | 29.2% | 34.1% | 30.2% | 0.21 |
| Lazio | 40.3% | 30.6% | 31.4% | 0.45 |
| Abruzzo | 80.0% | 27.8% | 20.2% | 0.81 |
| Molise | 32.0% | 25% | 34.4% | 0.29 |
| Campania | - | - | - | - |
| Puglia | 33.6% | 57.3% | 38.2% | 1.08 |
| Basilicata | 40.4% | 66.1% | 28.8% | 0.98 |
| Calabria | 24.4% | 29.3% | 31.3% | 0.06 |
| Sicilia | 45.4% | 59.9% | 37.7% | 1.37 |
| Sardegna | 17.8% | 40.0% | 22.1% | -0.30 |
