# Supplementary material for: Association between quality of governance, antibiotic consumption, and antimicrobial resistance: an analysis of Italian regions
Source: Antimicrob Resist Infect Control. 2023 Nov 21;12:130. doi: 10.1186/s13756-023-01337-6 (PMC10662482; doi:10.1186/s13756-023-01337-6)
Supplement: Supplementary file 3 — Additional file 3. Pillars and sub-pillars of the European Quality of Government Index across Italian regions in 2021 [file 13756_2023_1337_MOESM3_ESM.docx]

**ADDITIONAL FILES**

**Additional File 3. Pillars and sub-pillars of the European Quality of Government Index across Italian regions in 2021.**

| **Region** | **EQI** | **Quality** | **Impartiality** | **Corruption** | **Experienced corruption** | **Perceived corruption** |
| --- | --- | --- | --- | --- | --- | --- |
| Piemonte | -0.52 | -0.26 | -0.83 | -0.40 | 1.10 | 0.41 |
| Valle d'Aosta | -0.52 | 0.01 | -0.77 | -0.76 | 0.68 | 0.18 |
| Liguria | -0.63 | -0.50 | -0.83 | -0.49 | 1.01 | 0.34 |
| Lombardia | -0.81 | -0.24 | -1.22 | -0.89 | 0.60 | 0.00 |
| Autonomous Province of Bolzano | -0.25 | 0.32 | -0.73 | -0.32 | 0.75 | 0.93 |
| Autonomous Province of Trento | 0.01 | 0.66 | -0.35 | -0.28 | 0.94 | 0.80 |
| Veneto | -0.15 | 0.41 | -0.65 | -0.19 | 1.30 | 0.61 |
| Friuli-Venezia Giulia | -0.06 | 0.36 | -0.42 | -0.11 | 1.12 | 0.94 |
| Emilia-Romagna | -0.39 | 0.33 | -0.91 | -0.54 | 0.91 | 0.36 |
| Toscana | -0.36 | 0.03 | -0.63 | -0.44 | 0.84 | 0.60 |
| Umbria | -0.73 | -0.33 | -1.13 | -0.66 | 0.98 | 0.06 |
| Marche | -0.75 | -0.61 | -0.99 | -0.56 | 0.95 | 0.27 |
| Lazio | -1.21 | -1.09 | -1.24 | -1.16 | 0.49 | -0.37 |
| Abruzzo | -1.11 | -1.01 | -1.25 | -0.94 | 0.60 | -0.09 |
| Molise | -1.22 | -1.32 | -1.29 | -0.92 | 0.81 | -0.25 |
| Campania | -1.93 | -2.11 | -1.57 | -1.90 | -0.14 | -1.12 |
| Puglia | -1.35 | -1.39 | -1.41 | -1.09 | 0.90 | -0.65 |
| Basilicata | -1.44 | -1.32 | -1.64 | -1.20 | 0.76 | -0.72 |
| Calabria | -2.09 | -2.14 | -2.24 | -1.65 | 0.75 | -1.54 |
| Sicilia | -1.36 | -1.10 | -1.51 | -1.34 | 0.59 | -0.80 |
| Sardegna | -1.27 | -1.16 | -1.57 | -0.93 | 0.67 | -0.13 |
